# High Voltage Flyback Converter for Safety Indicators in Electrical Testing Laboratories

**DOI:** 10.3390/s26010270

**Published:** 2026-01-01

**Authors:** Alexandru Dalin Drăgoi, Septimiu Lica, Ioan Lie, Mihai-Vasile Popescu

**Affiliations:** Applied Electronics Department, Politehnica University Timișoara, 300 223 Timișoara, Romania; alexandru.dragoi2@student.upt.ro (A.D.D.); ioan.lie@upt.ro (I.L.); mihai-v.popescu@student.upt.ro (M.-V.P.)

**Keywords:** flyback topology, DC-DC converter, high voltage, small size converter, PCB design, high voltage testing, high voltage isolation, valley switching, galvanic isolation

## Abstract

High voltage (HV) test environments require dependable visual status indicators to maintain operator safety; however, directly supplying these indicators from HV sources introduces substantial electrical and operational hazards. This work addresses these challenges through the design and implementation of a compact Flyback DC–DC converter that provides galvanic isolation and a stable low-power output specifically intended for LED-based safety beacons. While utilizing Discontinuous Conduction Mode (DCM) and valley-switching to minimize thermal stress, the primary innovation of this design lies in the rigorous optimization of the isolation barrier and PCB architecture to meet HV safety standards (such as IEC 60950-1) within a minimal physical footprint. Transformer parameters were determined using analytical design procedures and subsequently verified by circuit-level simulations, which confirmed correct DCM operation as well as rapid startup behavior without output overshoot. A two-layer PCB was designed in accordance with IPC-2221B standard, with particular emphasis on minimizing parasitic effects and thereby improving overall performance. Experimental characterization demonstrated stable output regulation and a strong correlation between measured and simulated waveforms. The proposed system enhances safety in HV laboratory settings while achieving a compact form factor and supporting a wide input voltage range.

## 1. Introduction

Modern electronic systems—encompassing communication networks, computing platforms, transportation systems, and medical instrumentation—are fundamentally dependent on increasingly complex electrical circuits. Ensuring the reliability and operational safety of these circuits necessitates rigorous testing protocols, particularly in safety-critical sectors such as the automotive, aerospace, biomedical, and defense industries. Within these domains, HV testing plays a key role in verifying insulation integrity, identifying latent or incipient faults, and reproducing realistic operating and fault conditions under controlled environments [1]. The occurrence and relevance of HV phenomena in these systems arise from the requirement to supply electrical energy at levels adequate for their intended performance, which often entails elevated voltages and associated electric field stresses.

Consequently, specialized sensors and actuators deployed in HV testing environments are essential for the monitoring and control of electrical systems operating under extreme electrical stress. These devices must maintain reliable functionality while subjected to elevated electrical potentials, sometimes exceeding several kilovolts, in order to ensure accurate measurement acquisition and safe actuation during insulation integrity assessment, fault diagnosis, and performance characterization of power apparatus. Representative sensing devices include voltage transducers [2], current transducers [3,4], partial discharge detectors [5], and thermal sensors [6], whereas actuation elements may comprise switching devices [7], mechanical positioning systems for test configurations, or piezoelectric actuators [8]. Because these sensing components interface directly with HV circuits, they demand stringent galvanic isolation and robust protection schemes to mitigate electrical hazards and prevent degradation or corruption of measurement and control signals. As a result, auxiliary power-conversion stages, particularly isolated DC–DC converters, are indispensable for supplying the low-voltage electronics associated with these sensors and actuators, thereby enabling high-fidelity data acquisition and precise control while preserving operator safety and overall system integrity.

HV circuits, such as those referenced, present substantial hazards during experimental evaluation, including the risk of electric shock, excessive thermal loading, and potential damage to measurement or auxiliary equipment, necessitating rigorous hazard mitigation strategies. To reduce these risks, visual safety indicators—for example, LED-based beacon lamps triggered by voltage presence sensors, are commonly integrated into test benches to provide clear status information. According to the European Standard EN 50191 [9], the operation of electrical test installations requires clear status indication to prevent unauthorized access during energized states. Specifically, for test stations without automatic protection (barriers), EN 50191 mandates the use of active warning lights (signal lights) to indicate the “Operation” states.

Similarly, IEEE 510-1983 [10] standard recommends that warning lamps be used to isolate test areas where physical barriers are impractical. However, directly powering these mandatory indicators from the HV mains is prohibited due to the risk of arc-flash and insulation failure. Consequently, there is a critical need for compact, specialized, highly isolated DC-DC converters capable of stepping down variable HV inputs (up to 1000 V) to safe low-voltage levels for these beacons—a niche requirement not adequately met by standard commercial power supplies, which typically lack the parameters and necessary clearance/creepage or connectivity for HV safety compliance, being meant for the usual low-voltage applications.

The Flyback converter constitutes one of the most extensively utilized topologies in isolated SMPS. Owing to its versatility, this converter is applicable across a broad range of power levels and operating conditions [11]. Its operating principle is based on the intermittent storage of energy in a transformer during the switch conduction interval, followed by the transfer of this energy to the output during the switch-off interval. In contrast to conventional transformers, the Flyback transformer simultaneously fulfills the roles of a transformer and a magnetic energy storage element (inductor). This dual functionality enables the realization of galvanic isolation while facilitating the generation of multiple isolated output voltages with minimal additional circuitry [12,13,14]. Flyback converters are particularly suitable for powering distributed sensor nodes and low- to medium-power applications (typically up to approximately 250 W), where they offer advantages such as reduced component count, compact implementation, cost-effectiveness, and relatively simple control requirements.

DCM operation, utilized in this project, simplifies control and reduces switching losses, making it ideal for low-power sensor applications. However, it requires careful design to manage ripple currents and ensure stable output voltage to prevent interference with sensitive measurement equipment.

In recent years, research on Flyback converters has largely focused on maximizing efficiency and power density through soft-switching techniques. Prominent advancements include the ACF topology, which utilizes a clamp capacitor and an auxiliary switch to recycle leakage energy and achieve ZVS [15,16]. Similarly, advanced QR controllers with adaptive valley locking have been developed to minimize switching losses across varying loads [17,18,19]. While these topologies excel in consumer electronics where efficiency is the primary metric, they require complex high side driving circuits, additional magnetic components, and precise tuning of resonant tanks [12,13].

In the specific context of HV testing environments, however, such complexity can be detrimental. Auxiliary power supplies for safety indicators must prioritize robustness, high noise immunity, and minimal component count to reduce the probability of failure. Furthermore, standard commercial ACF or QR drivers are rarely rated for the extreme input voltage dynamics (up to 1000 V) encountered in this application. Therefore, this design innovates by reverting to a simplified DCM architecture, but optimizing it specifically for HV isolation, wide-input regulation, and compact safety compliance, rather than pursuing marginal efficiency gains through complex soft-switching control.

The isolated Zeta converter represents a relatively uncommon yet effective topology for DC–DC conversion. Structurally, it is derived from the non-isolated Zeta converter by substituting the first inductor with coupled inductors and then a transformer, thereby providing electrical isolation between the input and output stages. The Zeta converter operates based on a two-inductor (or transformer-based) energy transfer mechanism to capacitors, in which energy is stored in the magnetic field during the switch-on interval and released to the capacitors and load during the switch-off interval. In contrast to the Flyback converter, the Zeta topology contains an inductive and capacitive filter at the output, which leads to reduced output current and voltage ripples and lower EMI [12,13]. Nevertheless, the increased control complexity and reduced efficiency at high conversion ratios render this topology less attractive for HV, low-current applications.

To ensure safety and compliance, HV testing must adhere to internationally recognized standards. Key standards include:

- IEC 60060:2025—High Voltage Test Techniques: Defines procedures for dielectric tests using AC, DC, and impulse voltages [20].
- IEC 60950-1:2001/COR1:2002—Information technology equipment—Safety: Defines general requirements for the protection against electric shock, fire, and energy hazards in information technology equipment operating at HVs [21].
- IEEE 4-1995—Standard Techniques for High-Voltage Testing: Establishes general methods for HV testing and measurement techniques applicable to various types of apparatus, including AC, DC, and impulse voltages [22].

These standards ensure that HV equipment is tested under consistent, repeatable conditions, reducing the risk of failure and enhancing operational safety.

This paper presents the design and implementation of a compact Flyback DC-DC converter tailored for powering LED safety beacons integrated in HV testing environments. The converter offers galvanic isolation essential for protecting sensor data chains, supports a wide input voltage range, and maintains a stable output suitable for low-current applications. Its integration into test benches enhances safety monitoring without adding complexity or clutter.

The structure of this paper is organized as follows: Section 2 discusses the selection of the converter topology and provides insights into the developed system. Section 3 briefly introduces the Flyback converter topology, whereas Section 4 presents the controller architecture and its operating principles. Section 5 and Section 6 describe the transformer design methodology and the corresponding analytical calculations. Section 7 then addresses the dimensioning and design calculations of the key additional components, and Section 8 reports and analyzes the simulation results. Section 9 deals with PCB layout considerations crucial for HV isolation. Then, Section 10 presents experimental validation and testing procedures. Section 11 offers a discussion of the results obtained, including upgrade proposals and lessons learned. Finally, Section 12 provides the conclusions and outlines potential directions for future work and system improvements.

## 2. Materials and Methods

This study reports on the design, numerical simulation, and experimental validation of a HV DC-DC Flyback converter intended for the power supply of LED-based safety beacons in controlled test environments. The converter is specifically engineered to comply with stringent HV safety standards while maintaining a minimal footprint suitable for retrofit integration. To achieve this, DCM was selected to optimize the magnetic volume and ensure robust stability across extreme input voltage variations.

The selection of an isolated DC-DC converter was motivated by its ability to provide galvanic isolation between the input and output stages. Given that the input voltage can reach values up to an order of magnitude higher than the output voltage, complete isolation is critical to mitigate potential electrical stress, safety hazards, and interference issues affecting the DUT.

Two widely adopted isolated DC–DC converter topologies were analyzed for low-power applications to identify the most suitable architecture. The Flyback DC–DC converter, which is functionally analogous to the Buck–Boost converter, employs a transformer as the primary energy storage and transfer element instead of a single inductor. This topology is extensively utilized in low-power systems due to its capability to generate high output voltages at relatively low output currents and to accommodate a wide range of input voltages. By contrast, the ZETA DC–DC converter operates in a manner similar to an isolated Buck converter, making it appropriate for low-power conversion, but generally less suitable when a high output voltage is required. Considering the design specifications of providing a tightly regulated high output voltage at low current over a broad input voltage range, while minimizing component count to reduce failure probability in safety-critical scenarios, the Flyback topology is determined to be the most appropriate choice for the intended DC–DC converter implementation.

To achieve a regulated output voltage, a closed-loop control system with feedback is required. The converter employs the TI (Dallas, TX, USA) UCC28742 controller [23], selected to implement a good control scheme without complex auxiliary circuits. Its valley-switching capability is utilized primarily to minimize thermal dissipation and EMI generation, thereby allowing the system to operate reliably in a compact, sealed enclosure without active cooling. The feedback loop incorporates a TI (Dallas, TX, USA) TL431 shunt regulator [24] in conjunction with an optocoupler to regulate the output voltage. Transformer design parameters were derived from the specified input and output voltage ranges, switching frequency, and current constraints, with specific margins applied to accommodate HV transients. The magnetic core is implemented using Mn-Zn N97 ferrite in a TDK/EPCOS (Munich, Germany) EFD 25/13/9 geometry [25].

Circuit operation was analyzed using the SIMetrix/SIMPLIS (Version 9.10v) simulation environment [26], with particular emphasis on startup transients, current waveforms, and output voltage ripple. The simulation results verify operation in DCM and confirm the theoretical design methodology.

The PCB was designed with a rigorous adherence to IEC 60950-1 safety standard [21], including appropriate conductor spacing and the application of an acrylic conformal coating. Compliance with IPC-2221 [27] was ensured with respect to clearance and creepage distances. A two-layer board topology was implemented, incorporating galvanic isolation between the primary and secondary areas. The overall board dimensions were selected to be compatible with standardized enclosure formats.

Testing was conducted using an Elektro-Automatik (Viersen, Germany) EA-PSB 11500-60 high-voltage programmable power supply and a Tektronix (Beaverton, OR, USA) MDO3104 mixed-domain oscilloscope equipped with a 10× high-voltage probe. The converter was characterized over a range of input voltages and load conditions, and the LED beacon served as an indicator to verify the stability of the output.

The design is based on the safety principles specified in IEC 60950-1:2001/COR1:2002 [21], which defines requirements for protection against electric shock and fire hazards in HV equipment. No limitations are imposed on the availability of the materials or methods described.

Generative artificial intelligence tools were employed solely to support the editorial refinement and structural organization of the manuscript text. No AI-generated data, figures, or elements of experimental design were produced or utilized.

## 3. General Overview of the Flyback Topology

**Figure 1:**
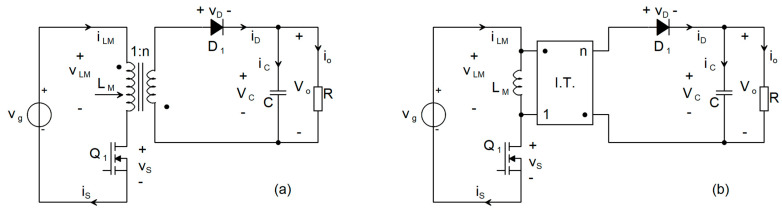
(**a**) Schematic of an ideal Flyback DC-DC converter [13]; (**b**) Simplified equivalent schematic of the Flyback converter.

Fundamentally, the Flyback DC-DC converter is derived from the Buck–Boost topology by substituting the single energy-storage inductor with a Flyback transformer [12,13]. As depicted in Figure 1a, the input voltage source V_g_ and the active switch Q_1_ (implemented as a FET) are connected in series with the primary winding of the Flyback transformer, whereas the passive switch D_1_ (a diode), the output capacitor C, and the load are connected in series with the secondary winding of the Flyback transformer.

For a detailed analysis of this topology, the Flyback transformer is modeled as a magnetizing inductance L_M_ connected in parallel with an ideal transformer, as illustrated in Figure 1b. The magnetizing inductance L_M_ exhibits behavior analogous to the energy-storage inductor in a conventional Buck–Boost converter [12].

The operating principle is analyzed under the assumption that both the inductance and the capacitance are sufficiently large. Under this condition, the converter operates in CCM, and the circuit alternates between two distinct topological states. DCM involves a third topological state, which is derived subsequently. In the first topological state, which has a duration denoted by t_ON_, the transistor Q_1_ conducts over a time interval t_ON_ = D·T_s_, while the diode D_1_ is reverse-biased. Here, D denotes the duty cycle of the PWM control signal applied to the transistor, and T_s_ = 1/f_s_ is the switching period corresponding to the switching frequency f_s_. During this interval, energy supplied by the input voltage source V_g_ is stored in the magnetizing inductance L_M_, resulting in the establishment of an electromagnetic field in the magnetic core.

In the second topological state (of duration t_OFF_), the transistor Q_1_ interrupts the current path between the input voltage source V_g_ and ground. Consequently, the energy previously stored in the magnetizing inductance L_M_ begins to decrease, inducing a current that is conducted by diode D_1_. During the time interval D’∙T_s_ = (1 − D)∙T_s_, the inductor L_M_ undergoes demagnetization, and the magnetic energy stored in the transformer is transferred to the load resistance R at the output, thereby also charging the capacitor C. Under these conditions, during the first topological state the output capacitor C sustains an approximately constant output voltage. Furthermore, the output voltage and current of the converter are scaled by a factor of 1:n with respect to the corresponding input quantities, where n denotes the transformer turns ratio.

### 3.1. The DC Mathematical Model of Flyback Converter in CCM Operation

**Figure 2:**
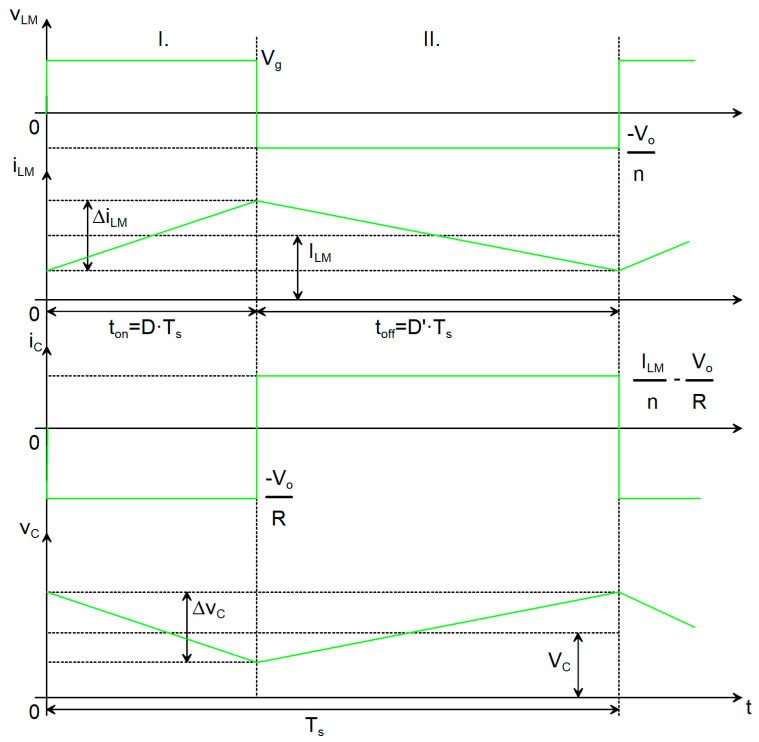
Waveforms relative to the reactive elements for an ideal Flyback DC-DC converter operating in CCM: voltage across L_M_, current through L_M_, current through C, voltage across C.

**Figure 3:**
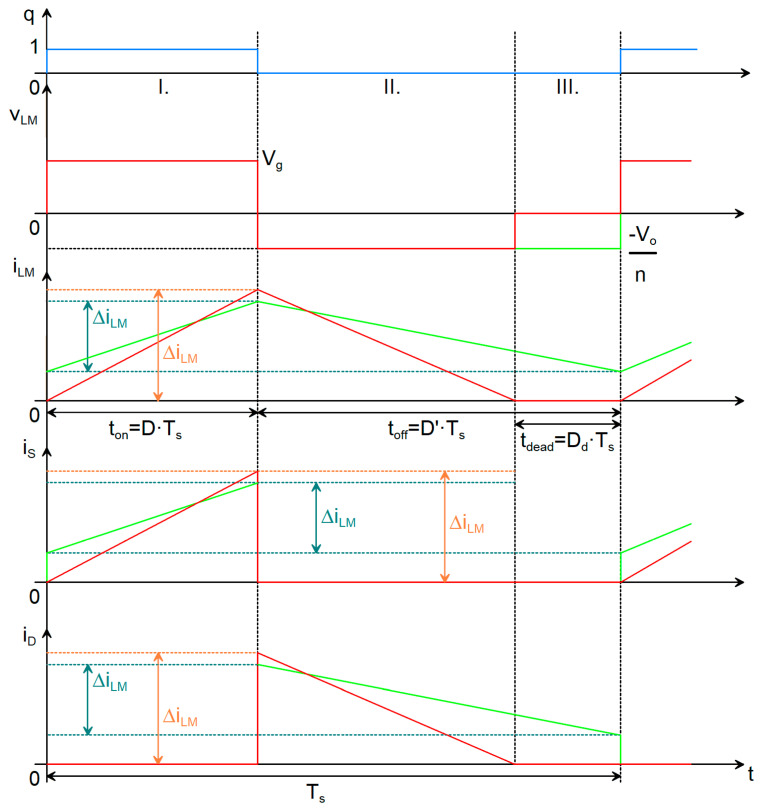
Waveforms of the currents through the switches and the inductor, comparison between CCM (green) and DCM (red): switching function, voltage across L_M_, current through L_M_, current through Q_1_, current through D_1_.

A PWM converter can operate in two distinct conduction regimes: CCM and DCM. When the active switch transitions from the off-state to the on-state before the magnetizing inductance current has completely decayed to zero, the inductor current remains strictly positive throughout the switching period. This operating condition is referred to as CCM. The corresponding ideal waveforms for a Flyback converter under CCM are illustrated in Figure 2 and are represented by the green traces in Figure 3.

In CCM, two distinct topological states are present during each switching period, with durations t_ON_ and t_OFF_, respectively. In the first topological state, the input voltage V_g_ is applied to the magnetizing inductance L_M_ through the conducting switch (transistor Q_1_). In the second topological state, the output voltage, scaled by the turns ratio n, is applied to the magnetizing inductance L_M_ via the ideal transformer, whose terminals are effectively reversed. Consequently, the voltage across L_M_ is negative in this interval. In this case, the voltage v_LM_ can be considered piecewise constant over each subinterval. Since the inductor current i_LM_ is related to the inductor voltage v_LM_ by the Faraday’s law, i_LM_ increases linearly during the first topological state and decreases linearly during the second. The current through L_M_ is piecewise linear. A defining characteristic of CCM is that the inductor current never falls to zero at any point in the switching cycle and that the diode conducts the whole t_OFF_ interval.

In the first topological state, the capacitor C is discharged through the load R. Under the assumption that the capacitance is sufficiently large and that the state variables exhibit small ripples [13], the current can be considered approximately constant during the interval t_ON_. During the interval t_OFF_, the capacitor C is recharged. With transistor Q_1_ in the blocking state, the freewheeling current i_LM_ flows through the ideal transformer, is scaled by the factor 1/n, and forward-biases diode D_1_, thereby supplying the output network R–C. The capacitor is thus charged by the difference between the inductor current conducted through the diode and the load (output) current. Consequently, the capacitor current i_C_ is piecewise constant.

As a result, the voltage across the capacitor decreases linearly during the first topological state and increases linearly during the second topological state, leading to a piecewise linear waveform. The amplitude of this variation corresponds to the output voltage ripple Δv_o_. Invoking the small-ripple assumption for the state variables, the quantities within each topological interval can be approximated by their respective DC values [13]. During the first topological state:

(1) $$v_{LM1}=V_{g}$$

(2) $$i_{C1}=-\frac{V_{o}}{R}.$$

Then, during t_OFF_ the quantities are:

(3) $$v_{LM2}=-\frac{V_{o}}{n}$$

(4) $$i_{C2}=\frac{I_{LM}}{n}-\frac{V_{o}}{R}.$$

In this analysis, I_LM_ denotes the average current flowing through the magnetizing inductance, whereas V_o_ represents the output voltage of the converter. Under steady state operating conditions, the output voltage is determined by the capacitor voltage V_C_, since the underlying energy transfer mechanism constrains the capacitor to sustain the load voltage. Accordingly, the relationship V_o_ = V_C_ holds. This equivalence is fundamental for converter modeling and control, as it establishes a direct correspondence between the principal energy storage element and the regulated output variable, thereby affecting both the transient (dynamic) response and the steady state voltage stability. By applying the volt-second balance principle [13] to L_M_, the capacitor voltage is derived, from which the static conversion ratio is subsequently obtained as:

(5) $$M_{CCM}\left(D\right)=\frac{V_{o}}{V_{g}}=n\cdot \frac{D}{\left(1-D\right)}.$$

Applying the charge balance principle [13] to C will help to calculate the current through the inductor:

(6) $$I_{LM}=\frac{n}{\left(1-D\right)}\cdot \frac{V_{o}}{R}=\frac{n^{2}\cdot D}{{\left(1-D\right)}^{2}}\cdot \frac{V_{g}}{R}.$$

These quantities can be used to calculate other parameters of the converter.

### 3.2. The Flyback Converter in DCM Operation

When the magnetizing inductance L_M_ is completely demagnetized, there exists a time interval during which the current through L_M_ falls to zero, at which moment both the transistor Q_1_ and the diode D_1_ are in the non-conducting state. This interval, referred to as the dead time, is given by t_DEAD_ = D_d_∙T_s_.

Under these conditions, the converter operates in DCM, such that the diode does not conduct over the entire t_OFF_ interval. The corresponding waveforms for this operating mode are shown in red in Figure 3.

As previously noted, the monotonic behavior of the inductive current i_LM_ is analogous to that observed in the other operating case. It is also observed that both the current and the voltage across L_M_ become zero during part of the switching period. This condition affects only the diode waveforms.

One advantage of operating this converter in DCM is the possibility of using a transformer of reduced size. However, DCM operation results in larger current ripples in the magnetizing inductor L_M_, which in turn leads to higher peak currents in the switching transistor, the diode, and the output capacitor.

Also, we may calculate the current through the capacitor I_C_ and the voltage across the inductor V_LM_ in all three topological states [12,13]. In topological state I (t_ON_):

(7) $$v_{LM1}=V_{g}$$

(8) $$i_{C1}=-\frac{V_{o}}{R}.$$

In topological state II (t_OFF_-t_DEAD_ = t_DEMAG_):

(9) $$v_{LM2}=-\frac{V_{o}}{n}$$

(10) $$i_{C2}=\frac{I_{LM}}{n}-\frac{V_{o}}{R}.$$

In the topological state III (t_DEAD_):

(11) $$v_{LM3}=0$$

(12) $$i_{C3}=-\frac{V_{o}}{R}.$$

By calculating the average voltage across the capacitor C, the dc conversion ratio of the converter operating in DCM is calculated:

(13) $$M\left(D\right)=\frac{V_{o}}{V_{g}}=n\cdot \frac{D}{1-D-D_{d}}.$$

The average current through the magnetizing inductance I_LM_ can be calculated as follows:

(14) $$I_{LM}=\frac{n}{1-D-D_{d}}\cdot \frac{V_{o}}{R}=\frac{n^{2}\cdot D}{{\left(1-D-D_{d}\right)}^{2}}\cdot \frac{V_{g}}{R}.$$

The peak-to-peak ripple of the current through the magnetizing inductance can be computed with:

(15) $$∆i_{LM}=\frac{D\cdot \left|v_{LM1}\right|}{L_{M}\cdot f_{s}}=\frac{D\cdot V_{g}}{L_{M}\cdot f_{s}}.$$

The ripple of the output capacitor voltage is given by:

(16) $$∆v_{c}=\frac{D\cdot \left|i_{c1}\right|}{C\cdot f_{s}}=\frac{D\cdot V_{o}}{R\cdot C\cdot f_{s}}.$$

The average voltage stress of the transistor is computed from the second topological state, when it is off:

(17) $$V_{s}=V_{g}+\frac{V_{o}}{n}=\left(1+\frac{M}{n}\right)\cdot V_{g}.$$

The dc current stress for the active switch is derived from the first topological state:

(18) $$I_{S}=D\cdot I_{LM}=\frac{n\cdot D}{1-D-D_{d}}\cdot \frac{V_{g}}{R}=M\cdot \frac{V_{g}}{R}.$$

For the diode, considering the state I, the voltage stress will be:

(19) $$V_{D}=n\cdot V_{g}+V_{o}=\left(M+n\right)\cdot V_{g}.$$

And finally, the dc current stress of the diode is calculated in state II:

(20) $$I_{D}=\left(1-D-D_{d}\right)\cdot I_{LM}=n\cdot \frac{V_{g}}{R}.$$

When high-quality components are employed, the ideal-case calculations exhibit close agreement with the actual circuit behavior. Under these conditions, the idealized analytical results can be used directly as the basis for designing a practical Flyback converter circuit.

## 4. Choosing the Controller

**Figure 4:**
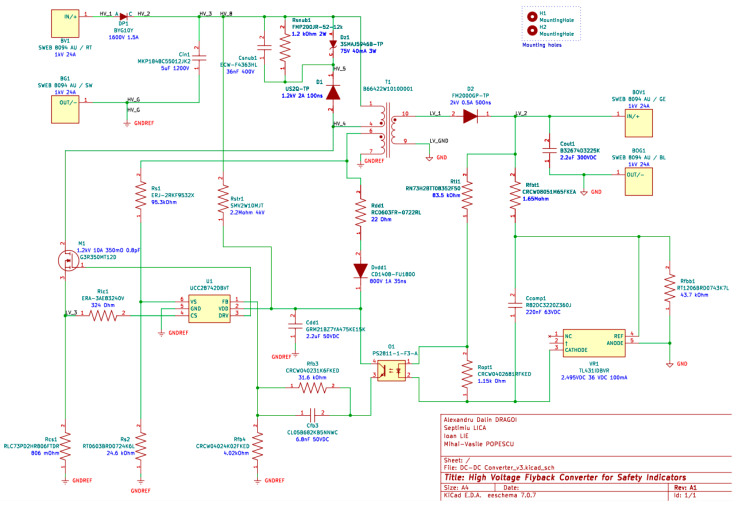
Schematic of the proposed circuit.

An IC is employed to generate the PWM signal that drives the power transistor. The selected controller is the TI UCC28742 [23], specifically designed for Flyback converters and low-power applications. It regulates a constant output voltage by means of an optocoupler and a shunt regulator within the feedback loop. Operating in DCM and utilizing valley-switching, the controller reduces switching losses and enhances overall efficiency [23,28]. It supports a switching frequency f_s_ range from 200 Hz to 80 kHz. Proper operation requires the inclusion of an auxiliary winding in the transformer. The corresponding application schematic is shown in Figure 4.

### 4.1. Start of the Conversion Cycle

The IC is connected to the power source through a startup resistor R_str1_, which charges the supply capacitor C_dd1_. Once the voltage across C_dd1_ reaches the IC startup threshold V_VDD(ON)_, the IC is activated and initiates switching of the FET for three cycles. If the input voltage V_g_ satisfies the minimum startup voltage required by the converter, continuous power conversion begins. Otherwise, the IC remains inactive until C_dd1_ discharges to the shutdown threshold V_VDD(OFF)_, at which point the startup sequence is reattempted. This sequence is repeated until V_g_ reaches the prescribed startup level. The capacitance of C_dd1_ must be selected such that it can reliably sustain IC operation and MOSFET gate drive during the detection and initial conversion intervals. To maintain stable operation during normal conversion, an auxiliary winding on the transformer supplies energy to C_dd1_, thereby ensuring the required bias current and voltage for the IC throughout the conversion cycle.

### 4.2. Output Voltage Regulation

**Figure 5:**
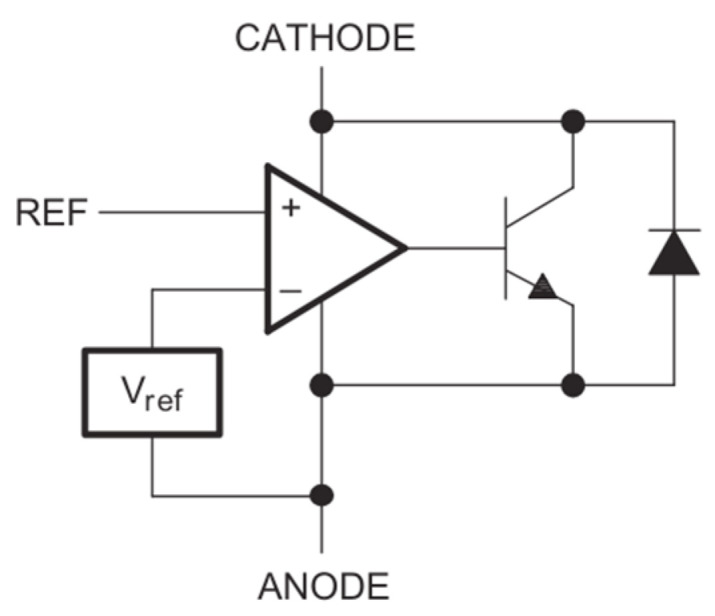
The equivalent schematic of the TL431 shunt regulator [24].

The output voltage is maintained at a constant level by means of a TL431 shunt regulator in conjunction with an optocoupler, both of which form integral components of the feedback loop. Figure 5 illustrates the internal architecture of the TL431, which integrates a precision V_ref_ = 2.5 V bandgap reference and an operational amplifier acting as a comparator [24]. In the proposed control strategy, this device functions as an error amplifier that drives the optocoupler LED, thereby closing the regulation loop across the isolation barrier.

The regulation mechanism operates as follows: A resistive divider (R_fbt1_, R_fbb1_) measures the converter output voltage V_o_. This divider is placed between the converter output and the secondary-side ground. If the regulator reference voltage (V_ref_ = 2.5 V) exceeds the voltage applied to the REF pin, the internal transistor remains in the off state, and the internal diode blocks current flow through the regulator. Conversely, if the output voltage V_o_ exceeds the target setpoint, the voltage at the REF pin rises above the internal V_ref_. The internal comparator amplifies this difference, causing the TL431 cathode voltage to drop and allowing more current to flow through the optocoupler LED.

This increased LED current is transferred across the galvanic isolation barrier to the phototransistor on the primary side. The phototransistor pulls more current from the controller’s FB pin.

The UCC28742 controller interprets this increase in FB current as a “light load” or “over-voltage” condition. Internally, this lowers the control law voltage V_CL_, which instructs the logic core to reduce the switching frequency or peak primary current, thereby reducing the energy transferred to the secondary side until V_o_ returns to the nominal level.

Contrarywise, if the output voltage drops (e.g., due to a load step), the TL431 restricts the LED current. This reduces the current drawn from the FB pin, causing V_CL_ to rise. The controller responds by increasing the switching frequency and duty cycle (up to the limits of the AM/FM modes described in Section 4.3), delivering more power to restore regulation.

To enhance the frequency response of the converter, a feedback capacitor C_comp1_ will be connected between the cathode and the reference pin of the regulator, thereby implementing a Type I compensation network. The resulting configuration will provide both current-mode and voltage-mode compensation, which in turn permits the use of simpler, lower-performance error amplifiers [13].

This converter is engineered to maintain a constant output voltage V_o_ until the load current reaches a predefined threshold. Once the output current surpasses the maximum design limit, the control scheme transitions to CC mode. In this operating regime, the output voltage can no longer be sustained at its nominal setpoint; as a result, the TL431 precision reference, in combination with the optocoupler, reduces the current at the FB pin to its minimum value. As the output load continues to increase under these conditions, the output voltage correspondingly decreases [23].

Under CC operation, the signals acquired from the CS and VSense pins enable accurate regulation of the output current I_o_, while the duty cycle D approaches its maximum value. In this operating condition, as the output voltage V_o_ decreases, the t_DEMAG_ interval increases. When the ratio between the magnetization period and the switching period (evaluated at the maximum switching frequency) reaches a predefined threshold established by the controller, the system attains the maximum permissible output current. Beyond this point, the controller triggers a protective shutdown within approximately 120 ms [23].

This control strategy ensures reliable operation under overload conditions, mitigates thermal stress on power semiconductor devices, and maintains conformity with established reliability standards. Moreover, the dynamic adaptation of the switching frequency f_s_ and t_DEMAG_ intervals underscores the critical role of robust feedback and monitoring mechanisms in attaining stable and predictable performance over a wide range of load conditions.

### 4.3. Control Law of the IC

**Figure 6:**
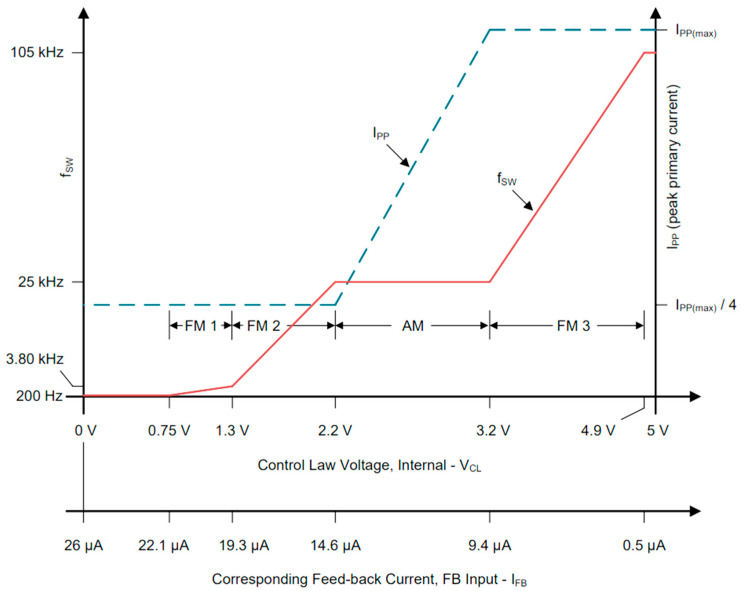
The control law of the IC [23].

The current applied to the FB pin influences the control voltage V_CL_ of the IC, which in turn adjusts the operating region, as revealed in Figure 6.

The IC control law uses both amplitude (AM) and frequency modulation (FM) to optimize the conversion efficiency based on the output load [23]. The four regions are:

- FM3: For high loads, the IC maximizes the frequency and current amplitude.
- AM: For moderate loads (10–90% of maximum), it maintains a 25 kHz frequency while adjusting the duty cycle.
- FM1/FM2: For light loads, it lowers the frequency and keeps the current amplitude at one-fourth of its maximum value.

These adjustments ensure efficient operation under different load conditions. The transistor will be switched on when a valley of the primary current occurs, specifically, when the drain to source voltage reaches a local minimum. This will lead to lower losses on the transistor.

### 4.4. Valley-Switching Technique

Since the converter operates in DCM, two distinct resonant networks are established during each switching period. The resulting resonant phenomena give rise to parasitic high-frequency oscillations, which can degrade the overall performance of the converter and adversely impact its EMC.

**Figure 7:**
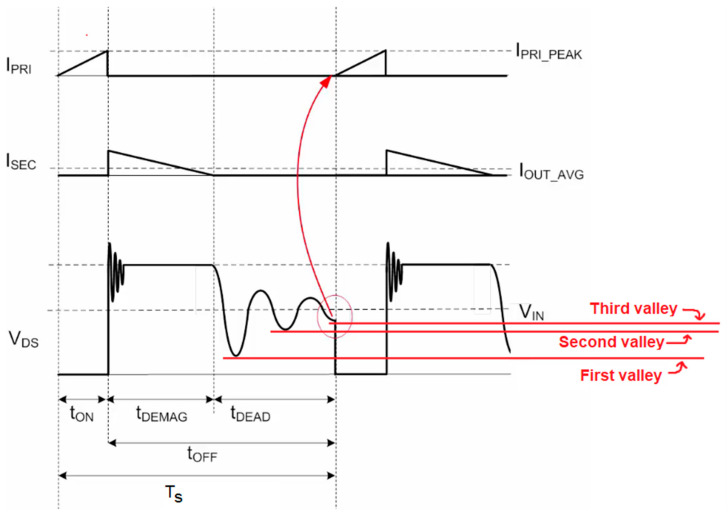
Waveforms for primary current, secondary winding current and voltage across the MOSFET in DCM operation, when valley-switching technique is employed. The turn on of the MOSFET is triggered when the third valley occurs [17].

The first resonant phenomenon arises at the instant when the switching devices are turned off. This resonance is predominantly induced by the interaction between the transformer leakage inductance and the parasitic capacitances of the MOSFET and the diode. As illustrated in Figure 7, at the beginning of the transformer demagnetization interval, t_DEMAG_, when the FET transitions to the off-state and ceases to conduct current from drain to source, a high-frequency parasitic oscillation is generated. These oscillations can result in increased switching losses, voltage overshoot, and elevated electrical and thermal stress on the semiconductor devices, thereby degrading overall reliability and efficiency.

As depicted in Figure 7, the second resonant circuit manifests during the third topological state, commonly designated as the resonance interval, t_DEAD_. This resonance is induced by the interaction between the transformer’s magnetizing inductance and the MOSFET parasitic capacitances. Provided that t_DEMAG_ is sufficiently long, the oscillation will decay naturally, such that the voltage at the end of this interval settles to a value equal to the converter input voltage.

Figure 7 additionally illustrates that this parasitic oscillation exhibits multiple local minima, commonly referred to as “valleys.” The controller utilizes a strategy known as valley switching, in which the MOSFET is turned on exclusively when the oscillating voltage reaches one of these minima. Valley switching may occur at any detected valley; if required, the controller dynamically adjusts the duration of the third topological state, t_DEAD_, so that turn-on coincides with the nearest valley. To preserve a constant output power level, the controller regulates the effective switching frequency by intentionally skipping one or more valleys between successive switching cycles [17].

In the present design, valley switching is employed over the entire output load range, or at least until parasitic oscillations have decayed. This strategy minimizes switching losses, mitigates EMI, and enhances overall conversion efficiency, with particularly pronounced benefits under light-load operating conditions, such as those considered here.

## 5. Determining Transformer Specifications: Calculation Method

The initial design parameters are summarized in Table 1. These are chosen based on the requirements of the light beacon and of the HV test-bench.

To determine the maximum duty cycle D_MAX_, the following parameters are required: the switching frequency f_s_, the DCM dead time t_DEAD_ and the maximum ratio between the demagnetization period t_DEMAG_ = t_OFF_ − t_DEAD_ and the switching period T_s_ (set internally by the IC). It is generally assumed that the resonance frequency is 500 kHz [23]. To begin, f_s_ = 67 kHz will be selected to ensure that the IC can operate in all regions. Then the needed formula is:

(21) $$D_{MAX}=1-\left(\frac{t_{DEAD}}{2}\cdot f_{s}\right)-D_{MAGCC}=0.458.$$

D_MAGCC_ is defined as the secondary diode conduction duty cycle when load current reaches a specified constant current limit operation. It is fixed in the IC at 0.475 [23]. With the knowledge of D_MAX_, the maximum ratio between the number of turns in the primary winding and the number of turns in the secondary winding of the transformer, N_PS(MAX)_, can be calculated:

(22) $$N_{PS\left(MAX\right)}=\frac{D_{MAX}\cdot V_{g\left(MIN\right)}}{D_{MAGCC}\cdot \left(V_{o}+V_{F}\right)}=0.5725,$$

and the forward voltage of the diode V_F_ is also taken into account. Then, the value of the current sense resistor R_cs1_, which the IC uses to detect the primary current, can be computed as:

(23) $$R_{cs1}=\frac{V_{CCR}\cdot N_{PS}}{2\cdot I_{oCC}}\cdot \sqrt{\eta_{TRAF}}=806\;m\Omega .$$

The value of η_TRAF_ can be set to 95%, if a 2.5% loss is estimated for the leakage inductance and another 2.5% loss for the core and windings. V_CCR_ represents the current regulation constant, obtained from the datasheet [23]. With the value of this resistor, the maximum primary current can be calculated:

(24) $$I_{P\left(MAX\right)}=\frac{V_{CST\left(MAX\right)}}{R_{cs1}}=0.955\;A,$$

where V_CST(MAX)_ represents the current detection threshold voltage on the CS pin, taken from the datasheet [23]. Now that these values are known, the inductance of the transformer’s primary winding can be designed as follows:

(25) $$L_{P}=\frac{2\cdot \left(V_{o}+V_{F}\right)\cdot I_{oCC}}{\eta_{TRAF}\cdot {I_{P\left(MAX\right)}}^{2}\cdot f_{s}}=436\;\mu H.$$

It is recommended that the minimum duration of the topological state I (t_ON_) be longer than the period t_CSLEB_ during which the IC checks for noise or resonance spikes that could cause false MOSFET activation [23]:

(26) $$t_{ON\left(MIN\right)}>t_{CSLEB}=195\;ns.$$

The calculated actual period is:

(27) $$t_{ON\left(MIN\right)Actual}=\frac{L_{P}}{V_{g(MAX)}}\cdot \frac{I_{P\left(MAX\right)}}{k_{AM}}=103.55\;ns<t_{CSLEB}.$$

The constant k_AM_ is taken from the datasheet [23]. Since t_ON(MIN)Actual_ is less than t_CSLEB_, the primary inductance of the transformer will need to be increased. Increasing the inductance will decrease the switching frequency f_s_. To ensure that t_ON(MIN)_ will always be greater than t_CSLEB_, L_P(Necessary)_ can be chosen as 2.7 mH:

(28) $$t_{ON\left(MIN\right)}=\frac{L_{P(Necessary)}}{V_{g(MAX)}}\cdot \frac{I_{P\left(MAX\right)}}{k_{AM}}=641.25\;ns.$$

The minimum ratio between the number of turns in the auxiliary winding and the secondary winding is:

(29) $$N_{AS\left(MIN\right)}=\frac{V_{VDD\left(OFF\right)}+V_{FA}}{V_{oCC}+V_{F}}=0.107,$$

based on the IC’s shutdown threshold voltage and taking into account the voltage loss across the auxiliary diode.

## 6. Transformer Design

### 6.1. Choosing the Core

In power electronics, ferrites constitute the most widely employed class of magnetic materials. Their electrical and magnetic properties depend strongly on the specific ferrite composition and alloying elements. For instance, Mn-Zn ferrites are typically used in applications with operating frequencies below 1 MHz, primarily due to their relatively low electrical resistivity (on the order of 1 Ω). Conversely, Ni-Zn ferrites, which exhibit significantly higher electrical resistivity (on the order of 10 kΩ), are preferred for applications operating at frequencies above 1 MHz [29].

Given that the selected IC supports switching frequencies up to 80 kHz, an Mn-Zn ferrite material was selected for the transformer core. In view of the converter’s voltage levels and operating frequency, the Mn-Zn N97 ferrite material was identified as a highly suitable choice. After the core material was specified, the core geometry and size were determined. Because the converter is required to exhibit a compact form factor, a core with minimal physical dimensions is preferred. Among the available small-core geometries, the EFD configuration was selected.

Ultimately, an EFD 25/13/9 core fabricated from N97 “ungapped” ferrite material was chosen [25]. This core is manufactured by TDK/EPCOS, which also provides a compatible THD coil bobbin for mechanical mounting of the core.

### 6.2. Transformer Design Calculations

The core parameters are obtained from the manufacturer’s datasheet [25] and presented in Table 2.

Given the value of the inductance per turn, the number of turns in the primary winding of the transformer can be calculated as follows:

(30) $$N_{P}=\sqrt{\frac{L_{P(Necessary)}}{A_{L}}}=35.85\;turns.$$

The secondary winding has:

(31) $$N_{S\left(MIN\right)}=\frac{N_{P}}{N_{PS\left(MAX\right)}}=62.88\;turns.$$

Considering that V_AUX_ should be approximately 18 V to keep the IC operational, the number of turns in the auxiliary winding will also be found by:

(32) $$N_{A}=\frac{V_{AUX}\cdot N_{s}}{V_{o}+V_{F}}=11.4\;turns.$$

Because the voltage is regulated by the closed loop, the number of turns may be approximated, as in Table 3. The turn ratio n will be 1.78. Given the maximum current ratings for the transformer windings, appropriate wire sizes can be selected (see Table 3).

Since the auxiliary winding conducts a comparatively low current, an AWG 26 conductor is likewise selected for this winding. This selection is additionally supported by the limited number of turns required and by the fact that the increased conductor diameter promotes a more effective utilization of the winding window at the base of the core support.

Kapton tape is employed to insulate both the transformer core and the outermost winding layers, thereby mitigating the risk of electrical breakdown and improving the overall mechanical robustness of the assembly.

Based on the data of the transformer, the operating parameters were computed: f_s_ ≅ 26 kHz and D_MAX_ ≅ 50%. The switching frequency is sufficient for the IC to operate in all the four regions (see Figure 6).

## 7. Design Calculations

The maximum blocking voltage for the diode in secondary is:

(33) $$V_{DR}=n\cdot V_{g\left(MAX\right)}+V_{o}=1878\;V.$$

In general, the peak amplitude of the resonant voltage across the leakage inductance V_LK_ can be estimated to be approximately 1 to 1.5 times greater than V_o_/n. This relationship is critical for predicting the voltage stress imposed on the switching devices, since excessive resonant amplitudes can result in overvoltage conditions, increased EMI, and potential long-term reliability issues. Therefore, accurate estimation of V_LK_ is highly beneficial for the design of snubber networks, the selection of MOSFETs with appropriate voltage ratings, and the assurance of adequate safety margins in isolated power converter topologies. The value for V_LK_ is:

(34) $$V_{LK}=1.5\cdot \frac{V_{o}}{n}=84.37\;V.$$

The voltage stress of the MOSFET is:

(35) $$V_{S}=V_{g\left(MAX\right)}+\frac{\left(V_{o}+V_{F}\right)}{n}+V_{LK}=1140\;V.$$

A 1.2 kV rated SiC MOSFET was selected.

To determine the specifications of the output capacitor C, the first step is to establish the maximum allowable ripple voltage Δv_o_. Since LED beacons have a defined turn-on threshold and the converter’s output voltage V_o_ is significantly higher than this threshold, a ripple of approximately ±1% at the output will not compromise the proper operation of the LEDs. Therefore, this ripple level can be considered acceptable for the design:

(36) $${\Delta v}_{o}=\pm 0.01\cdot V_{o}=\pm 1\;V.$$

This assumption streamlines the capacitor sizing procedure, as the ripple specification directly determines the minimum required capacitance.

In practical implementations, the output capacitor must supply sufficient energy storage to preserve voltage regulation over the switching period, while simultaneously attenuating high-frequency ripple components that may be transmitted to the load.

Furthermore, the capacitor selection process must explicitly consider the ESR, since ESR introduces additional ripple components and contributes to conduction losses. Hence, the design task becomes an optimization problem involving both the capacitance and the ESR, in order to satisfy the specified electrical performance constraints. A representative allocation of ripple contributions arising from ESR and from the filter capacitance can be formulated as follows:

(37) $$0.81\cdot {\Delta v}_{oR}=1.15\cdot {\Delta v}_{oC}=1\;V.$$

Then, the output filter capacitor C is designed with:

(38) $$R_{C\left(MAX\right)}=\frac{n}{I_{P\left(MAX\right)}}\cdot {\Delta v}_{oR}=2.31\;\Omega$$

and

(39) $$C=\frac{L_{P}\cdot {I_{P\left(MAX\right)}}^{2}}{4\cdot V_{o}\cdot {\Delta v}_{oC}}=2.77\;\mu F.$$

Using the datasheet [23], the remaining parameters required for proper IC operation can be promptly determined.

The passive components of the feedback loop are initially dimensioned by applying the pole–zero allocation method and are subsequently fine-tuned during the simulation phase.

The snubber resistance is estimated with:

(40) $$R_{snub}=\frac{{\left(V_{LK}+\frac{V_{o}}{n}\right)}^{2}}{\frac{1}{2}\cdot L_{LK}{\cdot f}_{s}\cdot {I_{P\left(MAX\right)}}^{2}\cdot \frac{V_{LK}+\frac{V_{o}}{n}}{V_{LK}}}=375.9\;k\Omega$$

and the snubber capacitance with

(41) $$C_{snub}=\frac{V_{LK}+\frac{V_{o}}{n}}{∆v_{snub}{\cdot f}_{s}\cdot R_{snub}}=1\;nF$$

The ripple voltage across the snubber capacitor is assumed to be 10% of its nominal DC value.

The RCD snubber network must be further optimized and fine-tuned on the practically realized PCB.

## 8. Simulations Results

**Figure 8:**
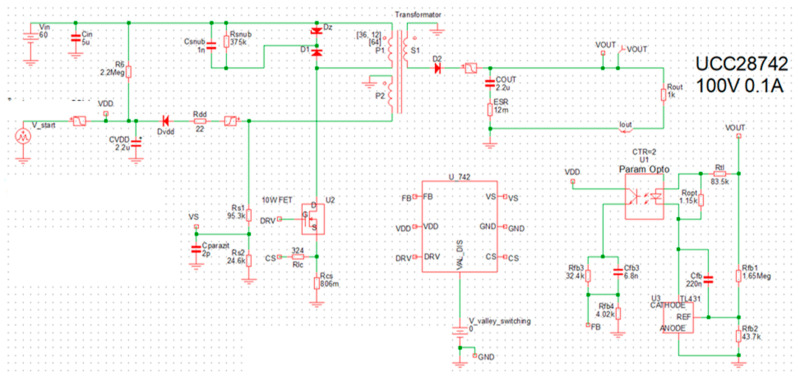
Circuit schematic implemented in the SIMetrix/SIMPLIS simulation program.

To simulate the circuit, the “SIMetrix/SIMPLIS” simulation software [26] will be used. The simulation schematic is reproduced in Figure 8. To ensure high fidelity between the simulation and the implemented prototype, a mixed-modeling approach was adopted. Passive components (resistors, capacitors and inductors) were modeled as ideal elements, with parasitic parameters such as ESR and transformer leakage inductance explicitly added based on the manufacturer datasheets. The active semiconductor devices (SiC MOSFET and HV diodes) were represented using PWL (piecewise linear) models that incorporate specific conduction parameters (R_DS(ON)_, V_F_) and junction capacitances (C_OSS_), enabling the accurate prediction of switching transitions and conduction losses.

**Figure 9:**
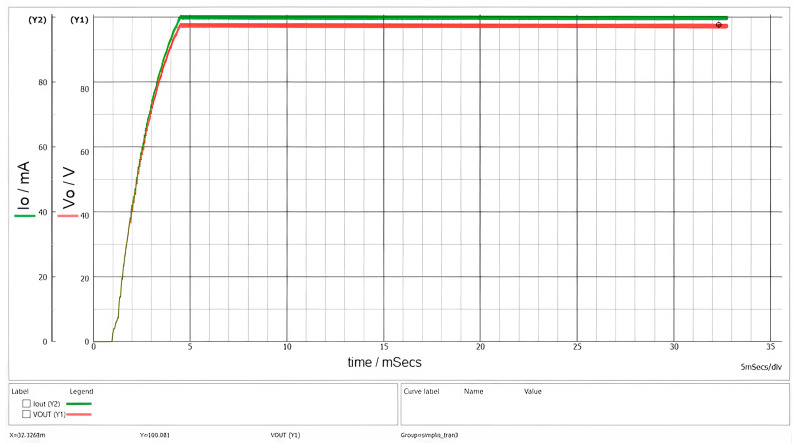
Waveforms of output current (green) and output voltage (red) with a 1 kΩ load and an input voltage of 60 V.

**Figure 10:**
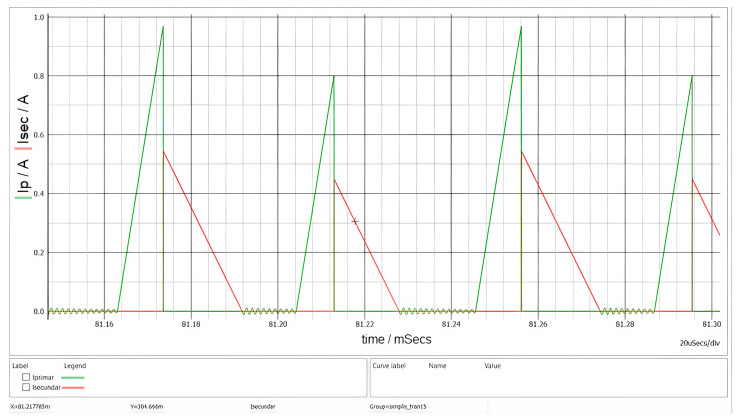
Waveforms of primary (green) and secondary (red) currents (R = 1 kΩ and V_g_ = 60 V).

In Figure 9, the startup sequence is presented, whereas Figure 10 illustrates the currents in the main windings of the transformer.

The designed converter exhibits a startup time of less than 5 ms and does not present any overshoot phenomena, as can be clearly observed in Figure 9. These characteristics render it well suited for supplying a beacon in optical signaling applications.

The green waveform in Figure 10 represents the magnetization of the magnetic core and the associated energy storage, while the red waveform corresponds to the demagnetization process and energy release. The triangular current waveforms and the presence of a dead time interval confirm that the converter operates in DCM. The oscillations observed during the dead-time interval t_DEAD_ arise from the parasitic elements of the MOSFETs and the PCB. These oscillations are attenuated by the snubber circuit, which is connected across the terminals of the primary winding. The final PCB capacitance is not known, that is why the fine-tuning stage is required on the prototype.

**Figure 11:**
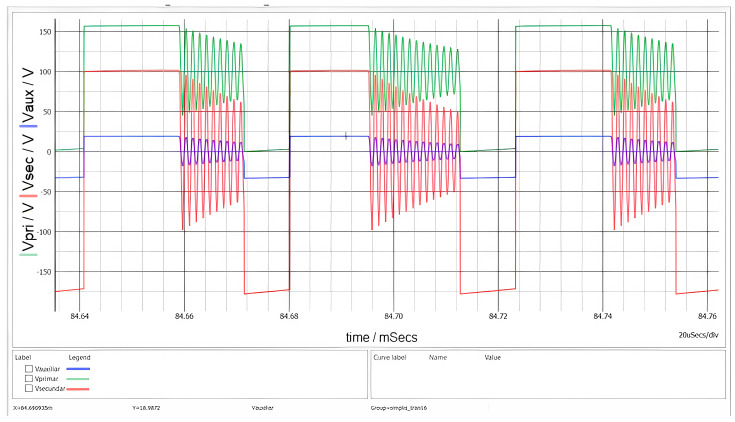
Waveforms of the primary (green), secondary (red), and auxiliary (blue) voltages when V_g_ = 100 V.

The voltages across the transformer windings are depicted in Figure 11. Two distinct oscillatory phenomena are observable, verifying the interaction between the circuit parasitic and the magnetic components.

At the instant of MOSFET turn-off, a high-frequency voltage spike and subsequent ringing are visible. This is caused by the resonance between the transformer’s leakage inductance and the MOSFET’s output capacitance. The implemented RCD snubber network effectively clamps the peak voltage, preventing breakdown.

Following the demagnetization interval t_DEMAG_, when the secondary current reaches zero, the voltage across the switch exhibits a lower-frequency oscillation. This behavior is characteristic of DCM operation and results from the energy exchange between the primary magnetizing inductance L_M_ and the total parasitic capacitance at the switch node.

During the conduction of the secondary diode (the flat plateau of the red trace), the voltage amplitudes are strictly determined by the transformer turns ratios. With an input voltage V_g_ = 100 V and a turns ratio of n ≅ 1.78, the reflected secondary voltage visible on the primary side corresponds to n∙(V_o_ + V_F_). On the other hand, the auxiliary winding voltage tracks v_SEC_ scaled by N_AUX_/N_SEC_. The simulation confirms these ratios, demonstrating that the cross-regulation between windings operates as designed.

**Figure 12:**
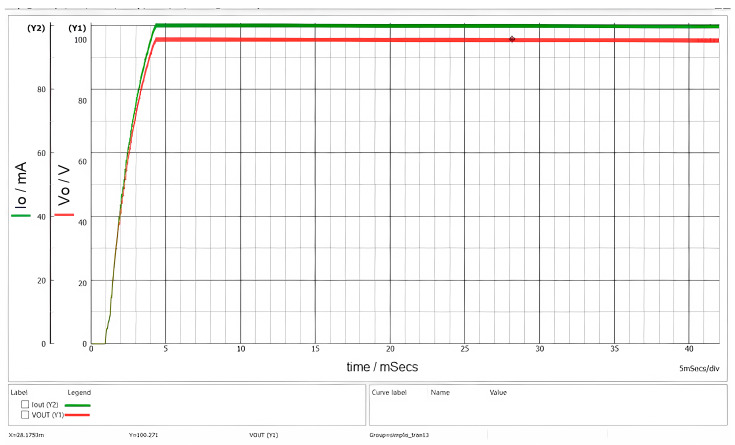
Waveforms of output current (green) and output voltage (red) with R = 1 kΩ and V_g_ = 1000 V.

Figure 12 illustrates the waveforms observed during startup for an input voltage V_g_ = 1000 V.

The tests with no load and overload also gave good results in simulation, proving that this SMPS will behave without issues in any of the practical situations.

## 9. Printed Circuit Board Design

Given that the converter operates at elevated HV levels, it is essential to maintain adequate clearance and creepage distances both between copper traces and between traces and pads, in order to minimize the probability of electrical arcing. The corresponding design constraints are defined in IPC-2221B [27]. The HV nets are explicitly identified in the schematic (see Figure 4), and dedicated layout rules are imposed for these connections. For an external layer of the PCB with solder-mask, the minimum spacing is specified as 3.05 mm for HV input traces (1000 V) and 0.4 mm for low-voltage traces (100 V). Furthermore, an acrylic conformal coating will be applied to the PCB to reduce the likelihood of surface discharge and to promote proper contact with the magnetic core.

For traces carrying currents up to 1 A, the conductor widths are dimensioned accordingly: 0.4 mm on the primary side and 0.2 mm on the secondary side. The overall PCB dimensions are constrained to be less than 96.3 × 48.5 mm, with an effective component placement area of approximately 3800 mm^2^, which provides sufficient space for component routing and placement. These dimensions are determined based on the availability of standard plastic enclosures. To satisfy the requirement for a minimal physical footprint, all resistors and capacitors are preferred to be implemented as SMDs. For the majority of these components, 0402 or 0603 packages are selected, as they provide an appropriate trade-off between compact size, electrical performance, and compatibility with automated assembly processes. In addition, the thermal behavior and voltage ratings of these components were analyzed to ensure adequate reliability under the converter’s specified operating conditions. The PCB stack-up consists of two electrical layers.

The revised design employs a single power resistor R_str1_ at the input, replacing the original series network of four trimming resistors. The primary-side current-loop traces are minimized in length and spatially isolated from other signals and power traces. Input and output interfaces are positioned on opposite edges of the printed circuit board and implemented using Schützinger (Stuttgart, Germany) HV banana connectors. The MOSFET gate-drive signal trace is routed orthogonally to the primary current-carrying traces and is shielded with ground to mitigate EMI.

Passive components associated with the FB pin, specifically the capacitors and resistors, are placed in close proximity to the IC to further reduce EMI susceptibility and loop area. The layout ensures complete galvanic isolation between the primary and secondary areas, with no conductive traces traversing the isolation barrier. In addition, the routing of the MOSFET control and auxiliary lines has been shortened to reduce parasitic capacitances and associated EMI emissions.

The implementation of Kelvin connections is essential in high-precision electronic circuits in which voltage drops along extended conductor traces can introduce non-negligible measurement errors or compromise the stability of feedback control loops. By segregating the sense and power conduction paths, the impact of parasitic series resistance is substantially mitigated, thereby enhancing the accuracy of the feedback signal and improving overall regulation performance. This methodology is particularly pertinent in isolated feedback topologies, in which an optocoupler functions as the primary signal-transfer element between the secondary and primary areas of the power converter.

**Figure 13:**
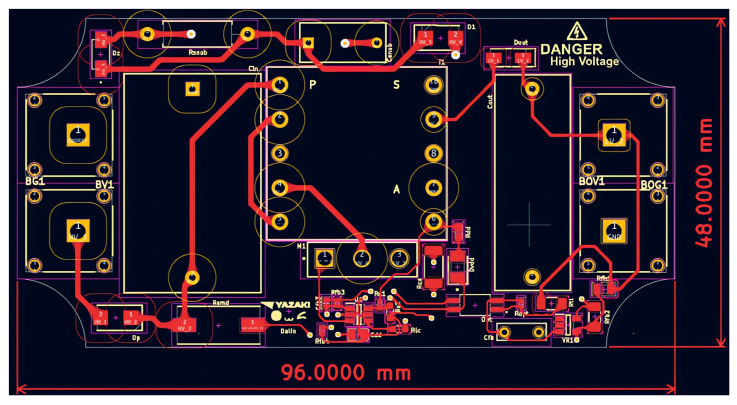
PCB—Top layer.

**Figure 14:**
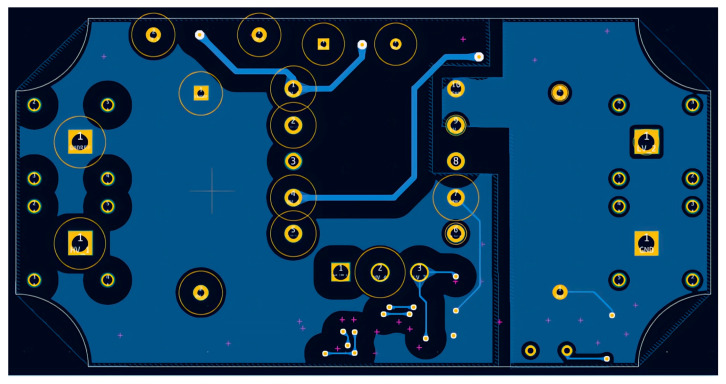
PCB—Bottom layer.

Furthermore, the implementation of copper pour islands as ground planes on both the primary and secondary sides reduce impedance, promotes more uniform current distribution, and enhances thermal dissipation. Collectively, these design measures result in improved electrical performance and diminished EMI. The corresponding PCB layouts artworks are shown in Figure 13 and Figure 14.

**Figure 15:**
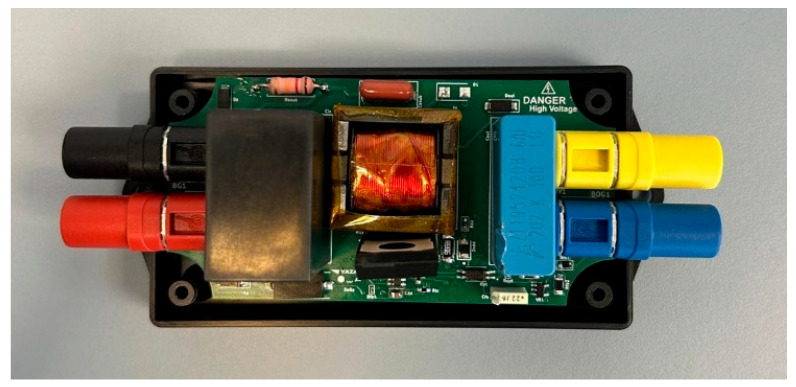
Populated PCB inside the enclosure.

The final assembly of the proposed product prototype is revealed in Figure 15.

Now the prototype is ready for practical testing.

## 10. Converter Testing

**Figure 16:**
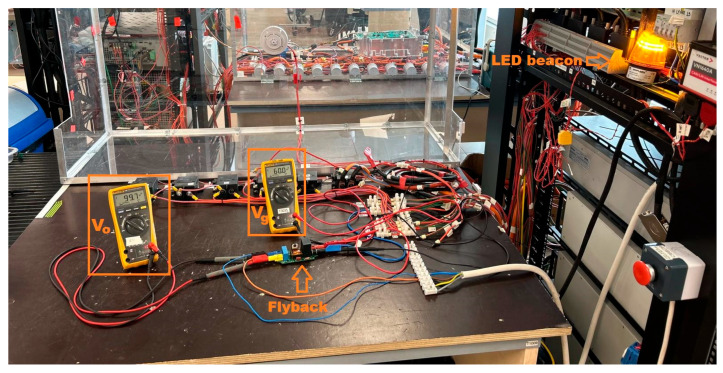
Converter tested at 60 V.

For the experimental characterization of the converter, a HV power supply (Elektro-Automatik, Viersen, Germany, type EA-PSB 11500-60), capable of delivering up to 1500 V and 60 A, will be employed. The converter will be positioned on the laboratory test bench and enclosed within a safety cage, as shown in Figure 16. Electrical measurements will be conducted using an oscilloscope (Tektronix, Beaverton, OR, USA, type MDO3104) in conjunction with a HV probe configured to a 10× attenuation setting. To ensure the rigor of the experimental validation, the potential sources of measurement uncertainty were qualitatively assessed. The primary contributors to uncertainty in the efficiency calculations are the accuracy tolerances of the DMMs (used for V_g_, I_g_, V_o_, and I_o_), particularly the burden voltage during low-current measurements. Regarding the thermal analysis, although the infrared camera was calibrated using a reference gray surface, variations in component emissivity and surface texture typically introduce an estimated uncertainty of ±2 °C. Furthermore, it is acknowledged that the HV oscilloscope probes introduce additional parasitic capacitance to the test node, which may result in slight deviations in the measured resonant frequencies and rise times compared, for example, to the ideal simulation models. Following completion of the measurement campaign, the LED beacon will be connected in order to evaluate the operational behavior under representative load conditions.

**Figure 17:**
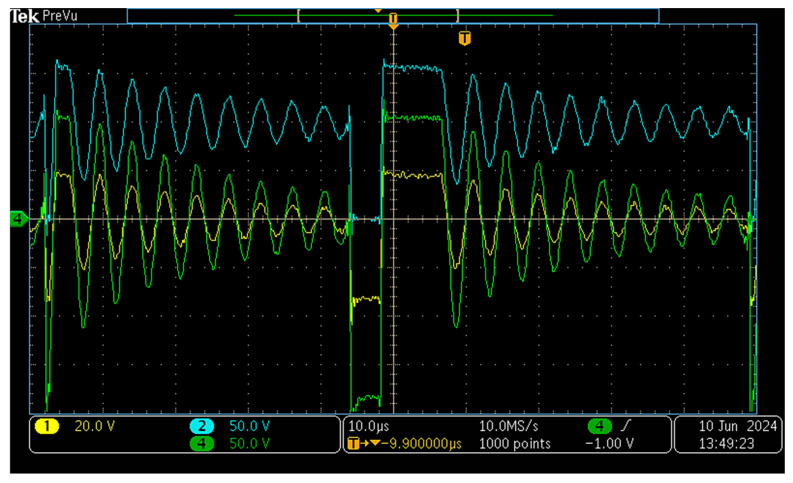
Waveforms of the v_S_ (blue), v_AUX_ (yellow) and v_SEC_ (green) voltages when the input voltage is 100 V.

An oscilloscope capture is presented in Figure 17. The measured waveforms closely correspond to those obtained in simulation, thereby confirming the correct operation and precise design of both the power converter and its control system. This experimental test was performed at V_g_ = 100 V, matching the conditions of the simulated waveform in Figure 11. The close agreement between the simulated and measured switching transitions validates the accuracy of the model. For further verification of operation across different input voltage levels, please refer to the Supplementary Videos.

**Figure 18:**
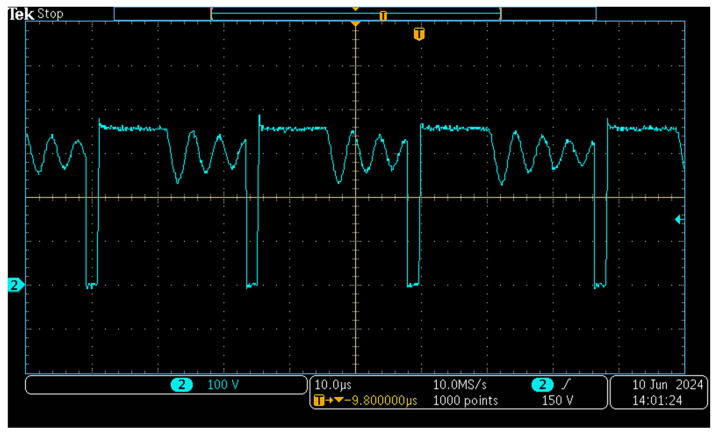
Waveform of the v_S_ voltages (blue) when the input voltage is 300 V.

In Figure 18 the voltage across the transistor is measured when the designed SMPS runs with an input of V_g_ = 300 V.

The characteristic damped oscillations associated with DCM operation are clearly observable during the interval in which the MOSFET remains in the off state.

**Figure 19:**
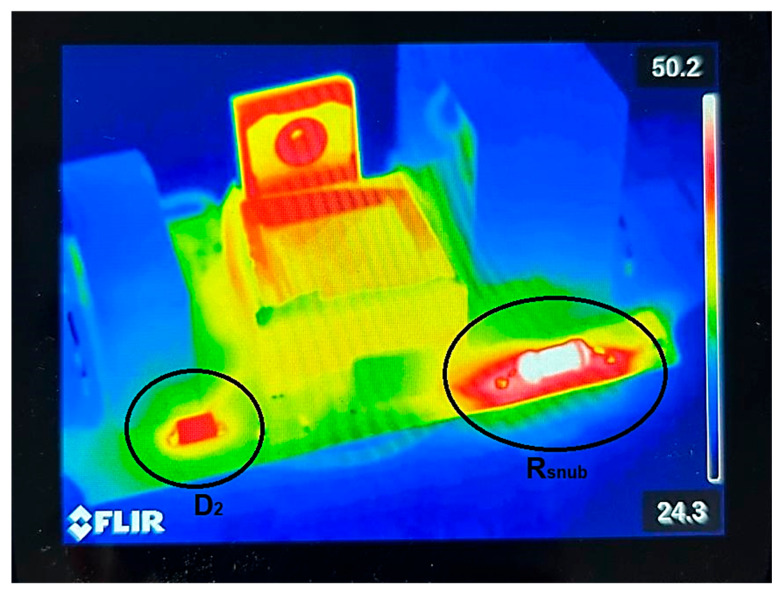
Temperature measurement through infrared photography of the prototype.

Thermal characterization constitutes a critical procedure for assessing the reliability and energy efficiency of power electronic systems. In this experiment, the converter was operated under multiple input voltage levels to emulate realistic operating conditions. After the system reached thermal steady state, infrared thermography was conducted to obtain the spatial temperature distribution of the PCB and its constituent components. The analysis revealed several localized regions of elevated temperature (hotspots), as illustrated in the reproduced image from Figure 19.

The results indicate that the MOSFET, the diode and the passive components associated with energy dissipation—specifically the snubber resistor R_snub1_ and the TL431 bias resistor R_tl1_—constitute the primary thermal hotspots in the system. Furthermore, diode D_2_ exhibited significantly elevated operating temperatures, which can be attributed to conduction losses and reverse-recovery-related dissipation. To quantitatively assess the reliability of the design, the temperatures measured via thermography were compared against the maximum rated operating temperatures of the key power-dissipating components. These values, summarized in Table 4, demonstrate that all critical components operate with substantial thermal margins. Even the hottest component, the snubber resistor, operates approximately 20 °C below its derating threshold, ensuring long-term stability without the risk of thermal runaway.

These observations underscore the necessity of robust thermal management measures, including optimized component placement, improved heat spreading, and the selection of low-loss semiconductors and passive devices, to enhance long-term reliability and mitigate the risk of thermal runaway in high-power-density converter architectures.

**Figure 20:**
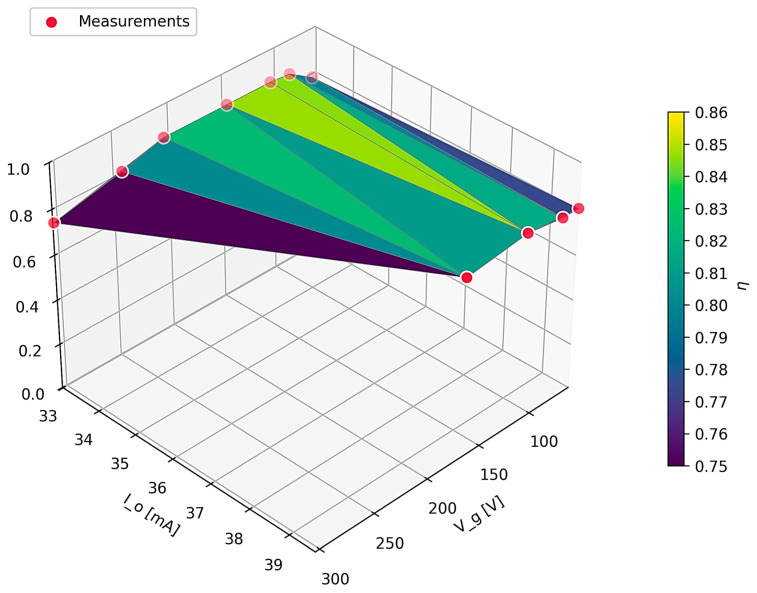
Experimental efficiency for variable input voltage and load.

To comprehensively evaluate the energy performance of the proposed converter, a ‘black box’ efficiency characterization was conducted. Precision DMMs were employed to simultaneously monitor the input parameters (V_g_, I_g_) and output parameters (V_o_, I_o_). The conversion efficiency (η) was subsequently derived from the ratio of the measured output power to the input power. A variable wire-wound power resistor served as the load; it is noted that this load introduces a non-negligible parasitic inductance, effectively emulating the reactive impedance characteristics (such as the inductance of long connecting cables) encountered in large-scale laboratory setups. The efficiency results, plotted across the operating range, are summarized in Figure 20. The converter demonstrated robust performance and stability under these reactive load conditions, achieving a maximum efficiency of 86.6%, while the minimum recorded efficiency was 74.45%. This efficiency mapping serves as a quantitative validation of the converter’s performance across several loads and input voltages, complementing the time-domain waveform analysis performed at some nominal setpoints.

The results demonstrate a high degree of congruence with theoretical predictions and other experimental results for a Flyback converter operating in DCM. The system achieves a peak efficiency of approximately 86% in the lower input voltage range (60 V ≤ V_g_ ≤ 100 V), where switching losses are minimized. As the input voltage increases toward 300 V, a monotonic decrease in efficiency is observed, reaching a minimum of approximatively 76%. This behavior is physically consistent with the topology’s characteristics, as switching losses in the MOSFET and power dissipation in the RCD snubber network increase proportionally with the square of the input voltage. The smooth and continuous nature of the efficiency surface confirms the stability of the control loop and the absence of erratic switching behavior across the tested envelope.

Furthermore, the prototype testing validated the system’s ability to withstand the full design input voltage range of 60 V to 1000 V, ensuring reliability under varying HV stress conditions.

Finally, simulations and measurements on the prototype prove the correct operation of the converter circuit.

## 11. Discussion

The primary objective of this study was to design and validate a compact, galvanically isolated HV Flyback DC–DC converter tailored for auxiliary power supply in safety-critical HV testing environments. The experimental and simulation results presented in the previous sections support the feasibility of the proposed topology operating in DCM.

The prototype demonstrated robust performance, maintaining a stable 100 V output across an input range of 50 V–300 V with a peak efficiency of 86.6%, decreasing to 76% at V_g_ = 300 V due to the expected increase in switching losses. Operation in DCM was confirmed to be advantageous for low-power applications, as anticipated, owing to the associated reduction in switching losses and the simplification of the control strategy. When compared to similar low-power flyback designs [12,13], which often prioritize efficiency at fixed loads, this system offers superior stability during the rapid startup transients (< 5 ms) required for safety signaling. The startup transient exhibited negligible overshoot. From a control theory perspective, this monotonic response confirms that the feedback loop possesses an adequate phase margin and is free from under-damped ringing, thereby serving as a time-domain validation of the system’s dynamic stability. Nonetheless, the inherent trade-off in DCM—namely higher peak currents and increased output ripple when compared with CCM—is consistent with trends previously reported for low-power isolated converters [12,13].

The implementation of Kelvin connections and copper-pour islands yielded quantifiable improvements in parasitic resistance and thermal performance. The primary advantage of this design is its ability to operate without an auxiliary power supply in HV environments, significantly reducing footprint. These layout strategies, which are widely endorsed in the field of power electronics, contributed to improved regulation accuracy and a reduction in EMI. However, the existing two-layer PCB architecture and the use of a dissipative snubber network imposes constraints on thermal dissipation and overall energy efficiency. Migration to a multilayer PCB design could provide additional suppression of EMI and enhanced signal integrity and efficiency, in accordance with recommendations outlined in IPC standards [27].

Circuit-level simulations conducted in SIMetrix/SIMPLIS supported the analytical design and verified DCM operation over the entire specified input voltage range. Time-domain waveform analysis confirmed the anticipated triangular inductor current profiles and clearly defined dead-time intervals, in accordance with established DCM operation theory. The voltage waveforms further validated the transformer design, as the measured steady-state plateaus across the primary, secondary, and auxiliary windings matched the theoretical values dictated by the winding turns ratios, confirming efficient magnetic coupling. Also, parasitic oscillations occurring during the demagnetization interval were effectively attenuated by the implemented snubber network, thereby underscoring the critical role of resonance damping in the design of HV power converters.

Experimental validation conducted under a real HV environment confirmed a high degree of congruence with simulation results, with the measured waveforms exhibiting a high degree of congruence with both theoretical and numerically simulated profiles. The converter consistently maintained a stable output voltage across a wide range of input voltages and load conditions, including overload regimes, without inducing premature activation of the protection circuitry.

Thermographic analysis demonstrated that the MOSFET, snubber resistor, TL431 bias resistor, and secondary rectifier diode constitute the dominant heat-generating elements under steady state operating conditions. This finding aligns with previously published studies, which report that switching devices and energy-dissipative components primarily govern the thermal distribution in Flyback converter topologies. The consequences are nontrivial: in the absence of appropriate thermal management strategies, these localized temperature elevations (reaching 50.2 °C) are likely to accelerate component degradation. This highlights a limitation of the current passive cooling approach in confined safety HV housings.

From a broader perspective, the proposed converter responds to a critical safety requirement in HV laboratories by providing galvanically isolated visual indication without introducing additional electrical hazards. Compared to traditional battery-powered beacons, this system eliminates maintenance overhead and hazardous waste, presenting a more sustainable industrial solution. These results further highlight the importance of isolated DC–DC conversion in HV environments and complement the existing body of work on SMPS design for safety-critical applications [20,21,22].

To benchmark the proposed design, the prototype was compared against commercially available ultra-wide input DC-DC converters and industrial reference designs utilizing similar SiC technology. As shown in Table 5, commercial modules (e.g., Mornsun PV15) and manufacturer evaluation boards capable of withstanding 1000 V inputs are predominantly designed for higher power, especially photovoltaic combiner boxes (DIN-rail mount) and typically occupy volumes exceeding 250 cm^3^. By tailoring the design specifically for low-power safety indicators (5–10 W) and optimizing the isolation barrier for this specific application, the proposed converter achieves a volume of only 195 cm^3^. This represents a 48% reduction in size compared to standard industrial counterparts, confirming its suitability for retrofit integration into space-constrained HV test setups.

Future work should explore:

- Advanced thermal management, such as incorporating heat spreaders, thermal vias, and GaN switching devices to minimize heat generation and improve component placement.
- EMI reduction by implementing a multilayer PCB and a shielded enclosure for compliance with stringent compatibility standards (to achieve Class B EMC compliance).
- Implementation of microcontroller-based digital control schemes, enabling adaptive frequency modulation to improve light-load efficiency and real-time fault diagnostics. Such approaches can enhance dynamic performance, improve efficiency under varying load conditions, and provide advanced protection mechanisms compared to traditional analog controllers.
- Long-term reliability assessment through accelerated aging tests under sustained HV stress is critical to validate the robustness of the proposed converter. These studies will provide insights into component degradation, thermal stability, and failure modes, ensuring compliance with industrial reliability standards and extending operational lifespan in demanding environments.
- Cost optimizations.

By addressing these areas, subsequent designs can achieve higher efficiency, improved reliability, and compliance with emerging safety and energy standards, thereby extending the applicability of this solution to broader industrial and research contexts. Finally, it must be noted that the scope of this work is strictly defined by the safety requirements of laboratory infrastructure. The proposed design is optimized specifically for low-power applications focused on visual signaling in HV test environments; consequently, the specific design trade-offs tailored to this niche should not be implicitly extrapolated to high-power or general-purpose energy conversion domains.

## 12. Conclusions

The design and implementation of the HV DC-DC converter represent practical solution for low-current HV applications. The system successfully achieved peak efficiency of 86.6% while maintaining robust regulation across the entire 60 V to 1000 V input voltage range. By efficiently converting elevated input voltages into a stable output voltage for devices such as LED beacons, this compact and mechanically robust converter contributes to enhanced safety within test environments. It effectively satisfies HV power requirements while reducing physical system complexity, wiring congestion and occupying a compact volume of only 195 cm^3^, thereby playing a critical role in enabling safe and efficient hardware testing. While the current design is limited by thermal constraints of the two-layer PCB, the integration of DCM operation provides a reliable, low-complexity control foundation. This work emphasizes the importance of reliable, application-specific power conversion solutions in strengthening safety protocols and improving operational efficiency across a range of high-reliability testing scenarios and industrial sectors. The converter is operated in DCM. Although CCM is generally preferred for many power conversion applications, DCM is particularly suitable and advantageous in low-power-demand scenarios due to its simplicity, inherent current limiting, and favorable behavior at reduced load conditions.

Future work may include the replacement of the existing transformer with a higher-performance design, refinement of the compensation network, active EMI filtering, GaN-based power stages to further increase power density and thermal margins, and the implementation of a multilayer PCB to mitigate EMI and enhance signal integrity. In addition, systematic optimization of component selection and power consumption will be conducted to comply with relevant energy-efficiency standards.

## Figures and Tables

**Table 1:** Design requirements.

Parameter	Notation	Value
Minimum input voltage	V_g(MIN)_	60 V
Maximum input voltage	V_g(MAX)_	1000 V
Output voltage	V_o_	100 V
Output current	I_o_	100 mA
Maximum output power	P_o(MAX)_	10 W
Maximum current in constant current mode	I_oCC_	120 mA
Output voltage when I_o_ = I_oCC_	V_oCC_	80 V

**Table 2:** EFD 25/13/9 made of N27 specifications (TDK).

Parameter	Notation	Value
Cross-sectional area of the core	A_Core_	58 mm^2^
Core volume	V_Core_	3310 mm^3^
Core permeability	μ_Core_	1640
Inductance per turn	A_L_	2100 nH

**Table 3:** Flyback transformer windings data.

Winding	Notation	Value	Maximum Current	Wire
Primary	N_P_	36 turns	I_P(MAX)_ = 0.95 A	AWG 26/0.14 mm^2^
Secondary	N_S_	64 turns	I_SEC(MAX)_ = 0.53 A	AWG 30/0.05 mm^2^
Auxiliary	N_A_	12 turns	Low current	AWG 26/0.14 mm^2^

**Table 4:** Temperatures of components.

Part	Notation	Part No.	Estimated Temperature	Maximum Allowed Temperature
Snubber resistor	R_snub_	RR02J1K2TB	48–50 °C	70 °C/155 °C * [30]
Rectifier HV diode	D_2_	FM2000GP	45–47 °C	150 °C [31]
SiC MOSFET	Q	G3R350MT12D	38–45 °C	175 °C [32]

Note: * For the selected resistor, power dissipation derating begins at 70 °C; at 155 °C, the maximum allowable power is reduced to 50%.

**Table 5:** Comparison with other ultra-wide input converters.

Parameter	Proposed Solution	Mornsun PV15-29B24 [33]	Microchip SiC Ref. Design [34]
Type	Custom Prototype	Commercial Module	Manufacturer Reference Design
Input Voltage	60–1000 V	200–1500 V	250–1000 V
Output Power	10 W	15 W	63 W
Topology	Flyback (DCM, SiC)	Flyback	Flyback (SiC)
Dimensions	97 × 50 × 40 mm	125 × 75 × 40 mm	153 × 62.2 × 40 mm
Volume	≅195 cm^3^	≅375 cm^3^	≅385 cm^3^
Target App.	HV Safety Beacons	Solar Energy Combiners	Aux. Supply for Solar Inverters

## Data Availability

Data, schematic diagrams and PCB for laboratory models are available on request. Please contact the corresponding author.

## Supplementary Materials

The following supporting information can be downloaded at https://www.mdpi.com/article/10.3390/s26010270/s1. Video S1: HV DC-DC converter operating at 300 V; Video S2: HV DC-DC converter operating at 500 V; Video S3: HV DC-DC converter operating at 910 V; Video S4: HV DC-DC converter tested at 100 V; Video S5: HV DC-DC converter tested at 200 V; Video S6: HV DC-DC converter tested at 300 V.

## Abbreviations

The following abbreviations are used in this manuscript:

AC	Alternating current
ACF	Active Clamp Flyback
A_Core_	Cross-sectional area of the ferrite core
AI	Artificial intelligence
A_L_	Inductance per turn for the magnetic core
AM	Amplitude modulation
AWG	American Wire Gauge
C	Capacitor (from the output of the converter)
CC	Constant-current (mode)
CCM	Continuous Conduction Mode
C_comp1_	Feedback compensation capacitor
C_dd1_	Filter capacitor for VDD (IC supply)
C_OSS_	MOSFET output capacitance
CS	Current sense
C_snub_	Snubber capacitor
D	Duty cycle of the PWM signal
DC	Direct current
DC-DC	DC to DC (conversion)
DCM	Discontinuous Conduction Mode
D_MAGCC_	Secondary diode conduction duty cycle when i_o_ reaches constant current set limit
D_MAX_	Maximum duty cycle
DMM	Digital multimeter
DUT	Device Under Test
D_1_	Diode 1
D’	Complementary of the duty cycle
EMC	Electromagnetic compatibility
EMI	Electromagnetic interference
ESR	Equivalent series resistance
FB	Feedback
FET	Field-effect transistor
FM	Frequency modulation
f_s_	Switching frequency
GaN	Gallium Nitride
HV	High-voltage
IC	Integrated circuit
i_C_	Current through C
i_C1_	Current through C in the 1st topological state
i_C2_	Current through C in the 2nd topological state
i_C3_	Current through C in the 3rd topological state
i_D1_	Current through the diode (current rectifier)
IEC	International Electrotechnical Commission
IEEE	Institute of Electrical and Electronics Engineers
I_g_	DC input current
I_LM_	DC current through L_M_
i_LM_	Current through L_M_
I_o_	DC output current
i_o_	Output current
I_oCC_	Maximum current in constant current mode
IPC	Institute of Printed Circuits, now Global Electronics Association
I_P(MAX)_	Maximum current through the primary winding
i_S_	Current through the active switch (transistor)
I_SEC(MAX)_	Maximum current through the secondary winding
k_AM_	AM control ratio
LED	Light emitting diode
L_LK_	Leakage inductance of the transformer
L_M_	Magnetizing inductance of a transformer
L_P_	Primary winding inductance
L_P(Necessary)_	Necessary primary winding inductance to meet the condition
M	Static conversion ratio in DCM
M_CCM_	Static conversion ratio in CCM
Mn	Manganese
MOSFET	Metal-oxide-semiconductor field-effect transistor
n	Transformer turn ratio
N_AS(MIN)_	Minimum transformer auxiliary to secondary turns ratio
N_P_	Primary winding number of turns
N_PS(MAX)_	Maximum transformer primary to secondary turns ratio
N_S(MIN)_	Secondary winding minimum no. of turns
PCB	Printed circuit board
P_o(MAX)_	Maximum required output power
PWL	Piecewise linear models from SIMPLIS
PWM	Pulse width modulation
q	Switching function, which controls the transistor
QR	Quasi-Resonant
Q_1_	Transistor 1
R	Resistor
R_cs1_	Primary winding current sense resistor
R_C(MAX)_	Maximum ESR of the capacitor
R_DS(ON)_	Drain to Source on-resistance of the MOSFET
REF	Reference (pin from the regulator)
R_fbt1_	1st resistor from the feedback voltage divider
R_fbb1_	2nd resistor from the feedback voltage divider
R_snub1_	Snubber resistance
R_str1_	Startup resistor
R_tl1_	Supply/bias resistor for TL431 IC
SiC	Silicon carbide
SMD	Surface mount device
SMPS	Switch-mode power supplies
TI	Texas Instruments (Inc.)
t	Time
t_CSLEB_	Leading-edge blanking time for the modulator
t_DEAD_	Dead time, when inductive energy is zero
t_DEMAG_	Demagnetization time
t_OFF_	Off-state time, when the transistor is blocked
t_ON_	On-state time, when the transistor conducts
t_ON(MIN)_	Minimum on-state time, when the transistor conducts
t_ON(MIN)Actual_	Minimum calculated on-state time, when the transistor conducts
T_s_	Switching period
v_AUX_	Voltage provided by the secondary winding
V_AUX_	Auxiliary voltage for the IC
V_C_	DC voltage across C
v_C_	Voltage across C
V_CCR_	Constant-current regulating level
V_CL_	Control law voltage
V_Core_	Ferrite core volume
V_CST(MAX)_	Maximum CS pin threshold voltage
V_DD_	Bias supply voltage
v_DS_	Drain to source voltage across the transistor
v_D1_	Voltage across the diode (current rectifier)
V_F_	Forward voltage drop of the diode
V_FA_	Forward voltage drop of the auxiliary diode
V_g_	DC input voltage source
V_g(MAX)_	Maximum input voltage
V_g(MIN)_	Minimum input voltage
V_LK_	Peak oscillation voltage due to resonance of leakage inductance
v_LM_	Voltage across L_M_
v_LM1_	Voltage across L_M_ in the 1st topological state
v_LM2_	Voltage across L_M_ in the 2nd topological state
v_LM3_	Voltage across L_M_ in the 3rd topological state
V_o_	DC output voltage
V_oCC_	Output voltage in constant current mode
V_ref_	Reference voltage from the regulator
V_S_	DC voltage across the active switch
v_S_	Voltage across the active switch (transistor)
v_SEC_	Voltage provided by the secondary winding
VSense	Voltage sense pin of the IC
V_VDD(OFF)_	IC’s VDD turn-off threshold
V_VDD(ON)_	IC’s VDD turn-on threshold
Zn	Zinc
ZVS	Zero Voltage Switching
Δi_LM_	Current ripples through L_M_
Δv_C_	Voltage ripples across C
Δv_o_	Output voltage ripple
Δv_oC_	Output voltage ripple by the limited filtering of capacitance
Δv_oR_	Output voltage ripple by ESR
Δv_snub_	Voltage ripple on the snubber capacitor
η	Converter efficiency
η_TRAF_	Transformer efficiency
μ_Core_	Ferrite core permeability
